# The New Zealand‐Dementia Prevention Research Clinics: Plasma biomarkers and the Alzheimer’s disease continuum

**DOI:** 10.1002/alz.093096

**Published:** 2025-01-09

**Authors:** Erin E Cawston, Leon Griner, Celestine YE Wong, Daniel J Myall, Nicholas J. Ashton, Fernando Gonzalez‐Ortiz, Kaj Blennow, Henrik Zetterberg, Joanna M Williams, Nicholas J Cutfield, Tim J Anderson, John C Dalrymple‐Alford, Lynette J Tippett, The New Zealand‐Dementia Prevention Research Clinics, New Zealand

**Affiliations:** ^1^ Centre for Brain Research, The University of Auckland, Auckland New Zealand; ^2^ University of Auckland, Auckland, Auckland New Zealand; ^3^ Centre for Brain Research, University of Auckland, Auckland New Zealand; ^4^ New Zealand Brain Research Institute, Christchurch New Zealand; ^5^ Department of Psychiatry and Neurochemistry, Institute of Neuroscience and Physiology, The Sahlgrenska Academy, University of Gothenburg, Mölndal, Gothenburg Sweden; ^6^ Institute of Neuroscience and Physiology, University of Gothenburg, Mölndal Sweden; ^7^ Department of Psychiatry and Neurochemistry, Institute of Neuroscience and Physiology, The Sahlgrenska Academy, University of Gothenburg, Mölndal Sweden; ^8^ Hong Kong Center for Neurodegenerative Diseases, Hong Kong China; ^9^ Dementia Research Centre, Department of Neurodegenerative Disease, UCL Queen Square Institute of Neurology, University College London, London, United Kingdom, London United Kingdom; ^10^ University of Otago, Dunedin New Zealand; ^11^ Brain Health Research Centre, University of Otago, Dunedin New Zealand; ^12^ Department of Medicine, University of Otago, Dunedin New Zealand; ^13^ Department of Medicine, University of Otago, Christchurch New Zealand; ^14^ University of Canterbury, Christchurch New Zealand; ^15^ School of Psychology, The University of Auckland, Auckland New Zealand

## Abstract

**Background:**

Single molecule array (Simoa) technology enables the detection of Alzheimer’s disease (AD) neuropathology in blood. This study compared cross‐sectional biomarker profiles for participants from the New Zealand‐Dementia Prevention Research Clinics (NZ‐DPRCs) who spanned the continuum from healthy older adults to a clinical diagnosis of AD.

**Method:**

NZ‐DPRC participants were clinically classified as cognitively unimpaired adults (CU, n=34), subjective cognitive decline (SCD, n=65), non‐amnestic mild cognitive impairment (single and multi‐domain, non‐aMCI, n= 23), amnestic MCI (single and multi‐domain, aMCI, n=104), and AD (n=27). A cross‐sectional analysis quantified nine plasma biomarkers using the HD‐X Simoa analyser (Quanterix, Billerica, MA). These were: amyloid‐beta peptides (Aß1‐40; Aß1‐42); total and phosphorylated tau species (total‐tau; brain‐derived tau (BD‐tau); p‐tau181; p‐tau217; p‐tau231); neurofilament light (NfL); and, glial fibrillary acidic protein (GFAP). All biomarkers except for Aß1‐40, Aß1‐42 and the Aß1‐42/Aß1‐40 ratio were natural log‐transformed to approximate normality and improve homogeneity of variance. Plasma biomarkers across the five groups (CU, SCD, aMCI, non‐aMCI and AD) were assessed by linear regression adjusted for age and sex and Tukey’s HSD post‐hoc test was used for pairwise comparisons between classification groupings.

**Result:**

This study included 253 participants from the NZ‐DPRCs (mean [SD] age, 69.8 [8.1] years, 131 females [52%], and 122 males [48%]). Biomarkers p‐tau217, GFAP, p‐tau181, Aß1‐42, BD‐Tau, Aß1‐42/Aß1‐40 ratio, p‐tau231 and NfL, all showed group differences (*F*(4,246); *P*<0.05; η^2^_p_ = 0.165, 0.109, 0.076, 0.073, 0.059, 0.058, 0.055, 0.044 respectively; Aß1‐40, *P*=0.06, η^2^_p_ = 0.035; total‐tau, *P*=0.67,η^2^_p_ = 0.010). Post‐hoc test for group comparisons showed significant differences (*P*<0.05) for GFAP between AD vs CU, SCD, non‐aMCI as well as SCD vs MCI; p‐tau181 for AD vs CU, SCD, aMCI, non‐aMCI; p‐tau217 for AD vs CU, SCD, non‐aMCI as well as aMCI vs CU and SCD; p‐tau231 for AD vs non‐aMCI; Aß1‐42 for aMCI vs CU and SCD; Aß1‐42/Aß1‐40 ratio and BD‐tau for SCD vs aMCI. ROC analysis will be reported for the diagnostic accuracy of these blood analytes.

**Conclusion:**

Simoa‐quantitated blood biomarkers in the NZ‐DPRC cohort showed utility for differentiating cross‐sectional groupings spanning the AD continuum.

